# New genetic data reveals a new species of *Zospeum* in Bosnia (Gastropoda, Ellobioidea, Carychiinae)

**DOI:** 10.3897/zookeys.1071.66417

**Published:** 2021-11-18

**Authors:** Thomas Inäbnit, Adrienne Jochum, Raijko Slapnik, Eike Neubert

**Affiliations:** 1 Institute for Biochemistry & Biology, University of Potsdam, Karl-Liebknecht-Strasse 24-25, House 26, 14476, Potsdam, Germany University of Potsdam Potsdam Germany; 2 Natural History Museum of the Burgergemeinde Bern, Bernastrasse 15, 3005, Bern, Switzerland Natural History Museum of the Burgergemeinde Bern Bern Switzerland; 3 Institute of Ecology and Evolution, University of Bern, Baltzerstrasse 6, 3012, Bern, Switzerland University of Bern Bern Switzerland; 4 Senckenberg Research Institute and Natural History Museum, Senckenberganlage 25, 60325 Frankfurt/M, Germany Senckenberg Research Institute and Natural History Museum Frankfurt Germany; 5 Drnovškova pot 2, Mekinje, SI - 1240 Kamnik, Slovenia Unaffiliated Kamnik Slovenia

**Keywords:** Dinarides, microsnails, molecular phylogenetics, shell variability, subterranean ecology, troglobitic microsnails

## Abstract

Recent integrative investigations of the terrestrial ellobiid genus, *Zospeum*, have revealed significant findings concerning its Alpine-Dinaric evolution and taxonomy. Due to the expected discrepancy between the useful, but limited, 1970s’ classification system based on shell data and the results of recent genetic analyses in the latest investigation, a revision of the entire radiation was undertaken, and a new classification system was devised by the present authors in an earlier paper. Concurrent to this work, molecular sequences from two Austrian caves were published independently of our revision by another research group. By incorporating these genetic data within our phylogenetic framework here, we show that the Austrian individuals are genetically most similar to *Zospeumamoenum* and consequently, classify them within that species. We additionally reveal two new genetic lineages from the largely under-sampled southern extension of *Zospeum’s* known distributional range. The first lineage, deriving from the region of Dubrovnik, Croatia, is a potential candidate for genetically clarifying *Zospeumtroglobalcanicum*. The second lineage derives from the municipality of Tomislavgrad, Bosnia-Herzegovina and is herein, described a new species: *Zospeumsimplex* Inäbnit, Jochum & Neubert, sp. nov.

## Introduction

The carychiid genus, *Zospeum*, consists of tiny (0.9–2.6 mm), troglobitic snail species that are distributed in two disjunct areas: a western zone, comprising the western Pyrenees and the Cantabrian mountains of Spain and France (Jochum et al. 2015a, 2019) and an eastern zone, encompassing the southeastern Alps and Dinarides of northeastern Italy, southern Austria, Slovenia, Croatia, Bosnia-Herzegovina and Montenegro (see Inäbnit et al. 2019). This work addresses the species rich, eastern radiation of *Zospeum.*

Until recently, the eastern radiation of *Zospeum* was largely classified using a scheme devised by Bole (1974), based solely on shell morphology. More recent studies (Weigand et al. 2011; Weigand et al. 2013; Jochum et al. 2015b), however, found Bole’s (1974) scheme, though effective for its time, now incongruent with genetic data, leading to a thorough revision by Inäbnit et al. (2019). They subdivided the eastern *Zospeum* radiation into 25 species that could be divided genetically into five species groups: the *Z.spelaeum* group (northeastern Italy, Slovenia, north-western Croatia; five species), the *Z.alpestre* group (Slovenian Alps and adjacent regions in Italy and Austria; four species), the *Z.obesum* group (southwestern Slovenia and adjacent Croatia; two species), the *Z.pretneri* group (Croatia, more or less close to the Adriatic coast; four species), and the *Z.frauenfeldii* group (southern Slovenia, northwestern Croatia, northwestern Bosnia-Herzegovina; five species); five species could not be assigned to any of the five groups due to lack of molecular data.

One of the issues raised in Inäbnit et al. (2019) is that *Zospeum*’*s* eastern distribution has been unevenly sampled throughout its history. Most studies covered almost only Slovenian (e.g., Frauenfeld 1854, 1856; Freyer 1855; Bole 1974; Weigand et al. 2013), Italian (Pezzoli 1992 and papers cited therein) and northwestern Croatian populations (Slapnik and Ozimec 2004; Inäbnit et al. 2019). The consequence of this sampling disparity is that we have very limited records from southern Croatia, Bosnia-Herzegovina and Montenegro (see Inäbnit et al. 2019: fig. 1a), none of which include genetic data. In fact, the only species described from the southern half of the *Zospeum*’s distribution range is *Zospeumtroglobalcanicum*Absolon 1916. Shells that obviously belong to different species exist in museum collections (see Inäbnit et al. 2019: fig. 10W-Z; Gittenberger 1975), but genetic data from these southernmost populations is still lacking for a contemporary, integrative taxonomic assessment. In the current study, we add new sequences from 12 specimens, collected in southern Croatia and Bosnia-Herzegovina to the existing genetic dataset.

**Figure 1.**
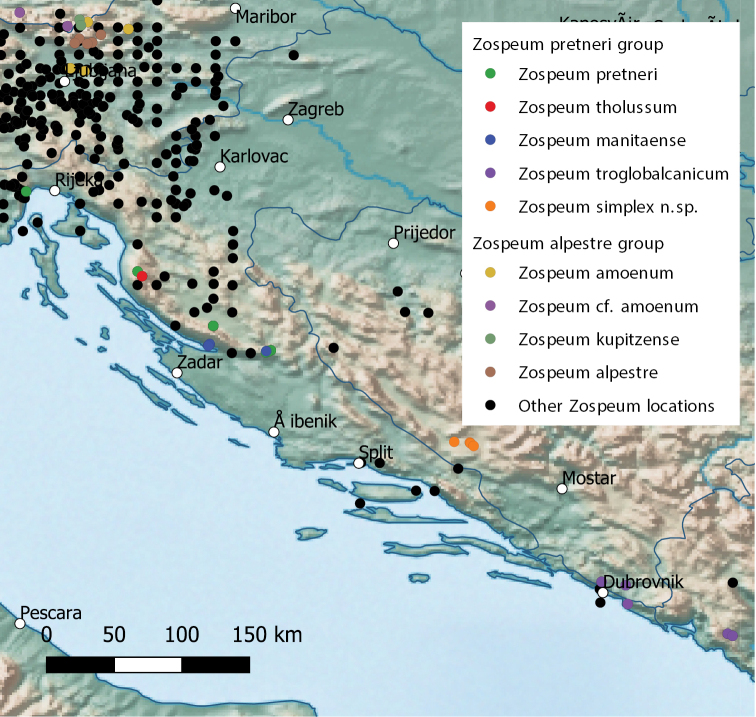
Map showing the distribution of the *Zospeumpretneri* group and the *Zospeumalpestre* group (except *Z.isselianum*). Austrian specimens from Kruckenhauser et al. (2019) are labelled as “Z.cf.amoenum”.

Approximately the same time as the revision by Inäbnit et al. (2019) was published, Kruckenhauser et al. (2019) published the results of a small barcoding study of specimens from Austria (for locations see Fig. 1). Due to this unfortunate overlap, their results could not be incorporated into the classification system proposed by Inäbnit et al. (2019). We have however, included these results in our work here.

## Materials and methods

Material is housed in the following collections:

**AJC** Adrienne Jochum Collection, Kelkheim, Germany;

**MCSMNH** Malacological Collection of the Slovenian Museum of Natural History (former CSR SASA, MZBI & SMNH) Ljubljana, Slovenia;

**NHMW**Naturhistorisches Museum Wien, Wien, Austria;

**NMBE**Naturhistorisches Museum der Burgergemeinde Bern, Bern, Switzerland;

**RSC** Rajko Slapnik Collection, Kamnik, Slovenia;

**SMF**Senckenberg Forschungsinstitut und Naturmuseum, Frankfurt am Main, Germany.

In order to preserve the shell from dissolution during the extraction, our DNA extraction protocol was based on a method initially described in Schizas et al. (1997) and partially modified after Böttger-Schnack and Machida (2011). DNA extraction was conducted on 12 ethanol-preserved individuals (NMBE 568052-568063). Each specimen was inserted into a 0.2-ml PCR-tube and dried at room temperature. Eight μl ddH_2_O and 2 μl 5× PCR-buffer (Promega 5× Colorless GoTaq Reaction Buffer) were added and the mixture was heated at 94 °C for 2 min. whereby 1.3 μl proteinase K solution (from the DNEasy Blood and tissue kit, Qiagen) were then added and the solution was homogenised and then incubated in a PCR-thermocycler at 55 °C for 15 min., afterwards at 70 °C for 10 min. The incubation was repeated once. Ten μl of Gene Releaser (Bioventures Inc.) were then added and the mixture was inserted into a thermocycler with the following protocol: 65 °C for 30 s, 8 °C for 30 s, 65 °C for 1.5 min., 97 °C for 3 min., 8 °C for 1 min., 65 °C for 3 min., 97 °C for 1 min., 65 °C for 1 min., 80 °C for 5 min. The mixture, including the intact shell, was centrifuged for 1 min. using a table centrifuge and the clear phase with the DNA was transferred to another 0.2 mL PCR-tube, where 15 μl of AE-Buffer (DNeasy Kit, Qiagen) was added. The shell was cleaned from the remains of the Gene Releaser chemicals by rinsing with 80% EtOH.

We used five markers, two mitochondrial (COI (658 bp), 16S (483 bp)) and three nuclear markers (H3 (330 bp), ITS2 (809 bp), 28S (590 bp)) with a total length of 2870 bp (for primers, see Table 1).

**Table 1.** Primers used in this study.

Marker	Primer Name	Primer sequence	Reference
COI	LCO1490 (F)	5‘-GGTCAACAAATCATAAAGATATTGG-3‘	Folmer et al. (1994)
COI	HCO2198 (R)	5‘-TAAACTTCAGGGTGACCAAAAAATCA-3‘	Folmer et al. (1994)
16S	16S F	5‘-CGGCCGCCTGTTTATCAAAAACAT-3‘	Palumbi et al. (1991)
16S	16S R	5‘-GGAGCTCCGGTTTGAACTCAGATC-3‘	Palumbi et al. (1991)
28S	LSU-2 (F)	5‘-GGGTTGTTTGGGAATGCAGC-3‘	Wade and Mordan (2000)
28S	LSU-4 (R)	5‘-GTTAGACTCCTTGGTCCGTC-3‘	Wade and Mordan (2000)
ITS2	ITS2ModA (F)	5’-GCTTGCGGAGAATTAATGTGAA-3’	Bouaziz-Yahiatene et al. (2017)
ITS2	ITS2ModB (R)	5’-GGTACCTTGTTCGCTATCGGA-3’	Bouaziz-Yahiatene et al. (2017)
H3	H3-F	5‘-ATGGCTCGTACCAAGCAGAC(ACG)GC-3‘	Colgan et al. (1998)
H3	H3-R	5‘-ATATCCTT(AGGGCAT(AG)AT(AG)GTG-3‘	Colgan et al. (1998)

The PCR-solution included the following admixture: 2 μl template, 12.5 μl GoTaq (Promega) polymerase, 8.5 μl of nuclease-free water, and 1 μl of both forward and reverse primer (10 μmol) respectively. In cases where the PCR signal was judged too weak, the reaction was repeated using 3 μl template DNA, 3 μl of the previous PCR product, and 5.5 μl of nuclease-free water. The amount of GoTaq and primers remained the same. The amplification was conducted using the following cycling protocols: For COI, the admixture was first heated up to 95 °C for 1 min, followed by 30 cycles of 30 s (of denaturation at 95 °C for 30 s, annealing at 52 °C for 30 s, extension at 72 °C for 1 min), and a final extension at 72 °C for 3 min. For 16S, the protocol started with 2:30 min at 90 °C, followed by 10 cycles of 30 s at 92 °C, 30 s at 44 °C, and 40 s at 72 °C, followed again by 30 s at 92 °C, 40 s at 48 °C, and 40 s at 48 °C. The protocol for 28S started with 1 min at 96 °C, then went into 35 cycles of 30 s at 94 °C, 30 s at 50 °C, and 1 min at 72 °C, finishing with 10 min at 72 °C. The ITS2 protocol started with 1 min at 96 °C, followed by 35 cycles of 30 s at 94 °C, 30 s at 44 °C, and 1 min at 72 °C, ending with 10 min at 72 °C. For H3, the admixture was first heated up to 95 °C for 3 min, followed by 40 cycles of 45 s at 94 °C, 45 s at 50 °C, and 2 min at 72 °C, finishing with 10 min at 72 °C. The protocols for COI and H3 could be used for both markers. The PCR products were sequenced at the LGC Genomics GmbH (Berlin, Germany) using their standard protocol.

Sequences received from LGC were imported into the Geneious 5.4.7 software (Kearse et al. 2012). The forward and reverse sequences for each gene and individual were combined and edited. In addition to the sequences that were generated during this study, we used the sequences previously used and generated in Inäbnit et al. (2019), as well as those generated by Kruckenhauser et al. (2019). The name of some of the Spanish specimens were updated based on the results of Jochum et al. (2019). A total list of samples can be found in Table 2. For each marker, sequences were aligned in Geneious using the MAFFT multiple sequence alignment plugin version 1.3.6 (based on MAFFT v7.308; Katoh et al. 2002; Katoh and Standley 2013), allowing the program to choose the most appropriate algorithm. The sequence length of each alignment was standardised to the length mentioned above.

**Table 2.** Specimens used in this study. Italicised accession numbers indicate sequences taken from BOLD, not italicised numbers are from GenBank.

Species	Source	Collection number	Locality	Coordinates	COI	16S	H3	28S	ITS2
*Carychiumtridentatum* (Risso, 1826)	Inäbnit et al 2019	NMBE 549936	Taunus, Eppstein, Germany	50.1601, 8.3846	MH383001	MH382969	MH383018	MH382989	MH383038
*Z.vasconicum* Prieto, De Winter, Weigand, Gómez & Jochum, 2015	Weigand et al. 2013	AJC 1875a	Cueva del Cráneo, Dima, Bizkaia, Spain	43.1287, -2.7348	*BARCA206-12*	KC206116	KC206249	—	—
	Weigand et al. 2013	AJC 1874b	Cueva Silibranka II, Manaria, Bizkaia, Spain	43.287, -2.945	*BARCA204-12*	KC206117	KC206248	—	—
	Weigand et al. 2013	AJC 1847c	Cueva de Ermita de Sandaili, Valle de Araotz, Bizkaia, Spain	42.9994, -2.4381	KM281092	KC206119	KC206247	—	—
Z.cf.schaufussi	Weigand et al. 2013	AJC 1878a	Cueva de Las Paúles, Monte Santiago, Castilla y León, Spain	43.1282, -2.7362	*BARCA194-12*	KC206113	KC206252	—	—
	Weigand et al. 2013	AJC 1844b	Cueva de los Cuervos, Barranco de Aranaga, Bizkaia, Spain	43.2829, -3.2588	*BARCA192-12*	KC206120	KC206246	—	—
*Z.praetermissum* Jochum, Prieto & De Winter 2019	Weigand et al. 2013; Romero et al. 2017	AJC 1842a	Cueva del Bosque, Inguanzo, Asturias, Spain	43.3123, -4.8724	KM281091	KC206121	KC206245	KM281051	—
*Z.zaldivarae* Prieto, De Winter, Weigand, Gómez & Jochum, 2015	Weigand et al. 2013	AJC 1876c	Cueva de Las Paúles, Monte Santiago, Castilla y León, Spain	43.1282, -2.7362	*BARCA209-12*	KC206114	KC206251	—	—
	Weigand et al. 2013	AJC 1876b	Cueva de Las Paúles, Monte Santiago, Castilla y León, Spain	43.1282, -2.7362	*BARCA208-12*	KC206115	KC206250	—	—
*Z.costatum* Freyer, 1855	Weigand et al. 2013	NMBE 553383	Jama 2 pri Jabljah, Loka pri Mengšu, Slovenia	46.1426, 14.5533	HQ171599	KC206158	KC206208	—	—
	Weigand et al. 2013	NMBE 553383	Jama 2 pri Jabljah, Loka pri Mengšu, Slovenia	46.1426, 14.5533	HQ171601	KC206159	KC206207	—	—
*Z.spelaeum* (Rossmaessler, 1838)	Weigand et al. 2013	NMBE 553316	Grotte Bac, Trieste Municipality, Trieste Prov., Italy	45.6361, 13.8717	*BARCA182-12*	KC206110	KC206255	—	—
	Weigand et al. 2013	AJC 1898a	Grotte Bac, Trieste Municipality, Trieste Prov., Italy	45.6361, 13.8717	*BARCA184-12*	KC206108	KC206257	—	—
	Weigand et al. 2013	NMBE 553316	Grotte Bac, Trieste Municipality, Trieste Prov., Italy	45.6361, 13.8717	—	KC206109	KC206256	—	—
	Weigand et al. 2013	NMBE 553314	Grotte d‘Ercole, near Gabrovizza, Trieste Prov., Italy	45.731, 13.7261	*BARCA181-12*	KC206111	KC206254	—	—
	Weigand et al. 2013	NMBE 553311	Velika Pasica, Gornji Ig, Slovenia	45.9189, 14.4934	*BARCA179-12*	KC206135	KC206231	—	—
	Inäbnit et al. 2019	NMBE 554396	Hotiške Ponikve, Hotičina, Slovenia	45.5735, 14.0309	MH382992	MH382954	MH383022	MH382974	MH383024
*Z.isselianum* Pollonera, 1887	Weigand et al. 2013	NMBE 553389	Turjeva jama, Robič, Kobarid, Slovenia	46.2435, 13.5046	HQ171594	KC206097	KC206268	—	—
*Z.amoenum* (Frauenfeld, 1856)	Inäbnit et al. 2019	RS 2037	Ihanščica, Ihan, Ljubljana, Slovenia	46.1216, 14.6476	MH383003	MH382971	MH383020	—	—
	Weigand et al. 2013	NMBE 553378	Konečka zijalka, Šmihel nad Mozirjem, Mozirje, Slovenia	46.4024, 14.9393	*BARCA123-10*	KC206179	KC206187	—	—
	Weigand et al. 2013	NMBE 553378	Konečka zijalka, Šmihel nad Mozirjem, Mozirje, Slovenia	46.4024, 14.9393	*BARCA124-10*	KC206178	KC206188	—	—
	Jochum et al. 2015	MCSMNH 40600a	Potočka zijalka, Olševa, Slovenia	46.4493, 14.6693	*BARCA211-13*	—	—	—	—
*Z.amoenum* (Frauenfeld, 1856)	Jochum et al. 2015	MCSMNH 40600a-2	Potočka zijalka, Olševa, Slovenia	46.4493, 14.6693	*BARCA212-13*	—	—	—	—
Z.cf.amoenum	Kruckenhauser et al. 2019	NHMW109000/AL/01821/8139	Steiner Lehmhöhle, Austria	46.42228, 14.53462	*AMOL570-19*	—	—	—	—
	Kruckenhauser et al. 2019	NHMW109000/AL/01821/8140	Steiner Lehmhöhle, Austria	46.42228, 14.53462	*AMOL571-19*	—	—	—	—
	Kruckenhauser et al. 2019	NHMW109000/AL/01822/8141	Hafnerhöhle, Austria	46.51200, 14.21623	*AMOL572-19*	—	—	—	—
	Kruckenhauser et al. 2019	NHMW109000/AL/01822/8142	Hafnerhöhle, Austria	46.51200, 14.21623	*AMOL573-19*	—	—	—	—
*Z.alpestre* (Freyer, 1855)	Weigand et al. 2013	NMBE 553391	Jama pod Mokrico, Kamniška Bistrica, Slovenia	46.3093, 14.5832	HQ171593	KC206099	KC206266	—	—
	Inäbnit et al. 2019	MCSMNH 40651a	Jelenska zijalka, Raduha, Slovenia	46.3656, 14.7567	MH383002	MH382970	MH383019	MH382990	MH383039
*Z.kupitzense* A. Stummer, 1984	Weigand et al. 2013; Romero et al. 2017	NMBE 553393	Ložekarjeva zijalka, Solčava, Slovenia	46.4268, 14.624	*BARCA125-10*	KC206150	KC206216	KM281049	—
*Z.exiguum* Kusčer, 1932	Inäbnit et al. 2019	NMBE 548774	Jama Borušnjak 3, Lupoglav, Ćićarija, Istra	45.3702, 14.1841	MH382994	MH382959	MH383009	MH382979	MH383030
	Weigand et al. 2013	NMBE 553384	Križna jama, Lož, Cerknica, Slovenia	45.7452, 14.4673	HQ171582	KC206162	KC206204	—	—
	Weigand et al. 2013	NMBE 553384	Križna jama, Lož, Cerknica, Slovenia	45.7452, 14.4673	HQ171585	KC206163	KC206203	—	—
*Z.obesum* (Frauenfeld, 1854)	Weigand et al. 2013	NMBE 553409	Krška jama, Krška vas, Slovenia	45.8899, 14.7711	*BARCA177-12*	KC206136	KC206230	—	—
	Weigand et al. 2013	NMBE 553409	Krška jama, Krška vas, Slovenia	45.8899, 14.7711	*BARCA175-12*	KC206137	KC206229	—	—
*Z.pretneri* Bole, 1960	Weigand et al. 2013	AJC 1370	Donja Cerovačka špilja, Kesići, Gračac, Croatia	44.2701, 15.8855	HQ171595	KC206151	KC206215	—	—
*Z.tholussum* Weigand, 2013	Weigand 2013	SMF 341633	Lukina jama – Trojama, Krasno, Croatia	44.7621, 15.0296	*BARCA120-10*	—	—	—	—
*Z.manitaense* Inäbnit, Jochum & Neubert 2019	Inäbnit et al. 2019	NMBE 548800	Manita peć, Starigrad, Croatia	44.311, 15.4792	—	MH382963	MH383012	MH382983	—
	Inäbnit et al. 2019	NMBE 548811	Manita peć, Starigrad, Croatia	44.311, 15.4792	MH383000	MH382968	MH383017	MH382988	MH383037
Z.aff.troglobalcanicum Absolon 1917	This work	NMBE 568052	Špilja Jezero, Cavtat, Konavle, Croatia	42.5858, 18.2569	MW786768	—	MW796484	MW784525	MW784537
	This work	NMBE 568053	Špilja Jezero, Cavtat, Konavle, Croatia	42.5858, 18.2569	MW786767	—	MW796485	MW784524	MW784536
*Z.simplex* sp. nov. Inäbnit, Jochum & Neubert	This work	NMBE 568054	Špilja Dahna, Omerovići, Bosnia and Herzegovina	43.6572, 17.2078	—	—	MW796475	—	—
	This work	NMBE 568055	Jama u kamenolomu, Cebara, Bosnia and Herzegovina	43.6517, 17.2133	MW786764	MW784509	MW796481	MW784526	MW784530
	This work	NMBE 568056	Jama u kamenolomu, Cebara, Bosnia and Herzegovina	43.6517, 17.2133	MW786765	MW784510	MW796478	MW784521	MW784532
	This work	NMBE 568057	Jama u kamenolomu, Cebara, Bosnia and Herzegovina	43.6517, 17.2133	MW786766	MW784511	MW796476	MW784520	MW784531
	This work	NMBE 568058	Jama u kamenolomu, Cebara, Bosnia and Herzegovina	43.6517, 17.2133	MW786763	MW784512	MW796477	—	MW784529
*Z.simplex* sp. nov. Inäbnit, Jochum & Neubert	This work	NMBE 568059	Vranjača, Grabovica, Bosnia and Herzegovina	43.6625, 17.1039	MW786762	MW784513	MW796486	MW784522	—
	This work	NMBE 568060	Jama Dobravljevac, Gornji Brišnik, Bosnia and Herzegovina	43.6347, 17.2328	MW786761	MW784515	MW796482	MW784527	MW784535
	This work	NMBE 568061	Jama Dobravljevac, Gornji Brišnik, Bosnia and Herzegovina	43.6347, 17.2328	MW786760	MW784516	MW796479	MW784523	MW784533
	This work	NMBE 568062	Jama Dobravljevac, Gornji Brišnik, Bosnia and Herzegovina	43.6347, 17.2328	MW786759	MW784514	MW796483	—	MW784534
	This work	NMBE 568063	Jama Dobravljevac, Gornji Brišnik, Bosnia and Herzegovina	43.6347, 17.2328	MW786758	MW784517	MW796480	MW784519	MW784528
*Z.subobesum* Bole, 1974	Weigand et al. 2013	NMBE 553326	Tounjčica, Tounj, Ogulin, Croatia	45.2439, 15.3253	HQ171602	KC206152	KC206214	—	—
	Weigand et al. 2013	NMBE 553326	Tounjčica, Tounj, Ogulin, Croatia	45.2439, 15.3253	HQ171604	KC206153	KC206213	—	—
	Weigand et al. 2013	NMBE 553328	Jopićeva špilja, Brebovnica, Krnjak, Karlovac, Croatia	45.2951, 15.5939	*BARCA172-12*	KC206125	KC206241	—	—
*Z.frauenfeldii* (Freyer, 1855)	Weigand et al. 2013	NMBE 553388	Podpeška jama, Podpeč, Dobrepolje, Slovenia	45.8393, 14.6863	HQ171587	KC206160	KC206206	—	—
	Weigand et al. 2013	NMBE 553388	Podpeška jama, Podpeč, Dobrepolje, Slovenia	45.8393, 14.6863	HQ171589	KC206161	KC206205	—	—
	Inäbnit et al. 2019	NMBE 548771	Hrustovača špilja, Hrustovo, Sanski Most, Bosnia and Herzegovina	44.6607, 16.7285	—	—	MH383006	MH382976	MH383027
*Z.bucculentum* Inäbnit, Jochum & Neubert 2019	Inäbnit et al. 2019	NMBE 548801	Jama na Škrilama, Netretić, Croatia	45.5277, 15.3476	MH382997	MH382964	MH383013	MH382984	MH383033
	Inäbnit et al. 2019	NMBE 548772	Pivnica špilja, Žakanje, Croatia	45.6108, 15.3617	—	MH382957	MH383007	MH382977	MH383028
	Inäbnit et al. 2019	NMBE 548806	Vrelić špilja, Donje Dubrave, Ogulin, Croatia	45.3114, 15.352	—	MH382966	MH383015	MH382986	MH383035
*Z.pagodulum* Inäbnit, Jochum & Neubert 2019	Inäbnit et al. 2019	NMBE 548805	Kučka jama, Lovran, Učka, Istra, Croatia	45.2985, 14.2135	MH382998	MH382965	MH383014	MH382985	MH383034
	Inäbnit et al. 2019	NMBE 548807	Grnjača špilja, Lovran, Učka, Istra, Croatia	45.2835, 14.2381	MH382999	MH382967	MH383016	MH382987	MH383036
*Z.robustum* Inäbnit, Jochum & Neubert 2019	Inäbnit et al. 2019	NMBE 554397	Tonkovića špilja, Ogulin, Croatia	45.3359, 15.2541	—	MH382953	MH383004	MH382973	MH383023
	Inäbnit et al. 2019	NMBE 548773	Budina špilja, Studenci, Croatia	44.7121, 15.3639	MH382993	MH382958	MH383008	MH382978	MH383029
	Inäbnit et al. 2019	NMBE 548777	Markov ponor, Lipovo polje, Croatia	44.7606, 15.1797	MH382995	MH382961	MH383010	MH382981	MH383032
	Inäbnit et al. 2019	NMBE 548787	Markov ponor, Lipovo polje, Croatia	44.7606, 15.1797	MH382996	MH382962	MH383011	MH382982	—
	Inäbnit et al. 2019	NMBE 548776	Vrlovka, Kamanje, Croatia	45.6319, 15.3934	—	MH382960	—	MH382980	MH383031
	Inäbnit et al. 2019	RS 2210a	Vrlovka, Kamanje, Croatia	45.6319, 15.3934	—	MH382972	MH383021	MH382991	MH383040
	Inäbnit et al. 2019	NMBE 554399	Židovske kuće, Cerovica, Žumberak, Croatia	45.8, 15.48	—	MH382955	—	MH382975	MH383025
	Inäbnit et al. 2019	NMBE 554400	Pušina jama, Jezemice, Žumberak, Croatia	45.7369, 15.3606	—	MH382956	MH383005	—	MH383026

Topologies were estimated using two different phylogenetic methods: Maximum Likelihood (**ML**) and Bayesian Inference (**BI**). The five markers were set as partitions in both of these methods, using a distinct model for the third codon in protein-coding genes (COI, H3). The maximum likelihood (ML) topology was estimated using the RAxML 7.2.8 (Stamatakis 2014) plugin of Geneious with the GTR gamma nucleotide model and 1000 bootstrap replicates. An additional ML tree was calculated for the *Z.pretneri* group (with *Z.robustum*NMBE 548777 as an outgroup) without H3 and 28S.

The Bayesian tree was reconstructed with MrBayes 3.2.6 (Huelsenbeck and Ronquist 2001) using the substitution models suggested by PartitionFinder (Lanfear et al. 2016, Lanfear et al. 2012, Guindon et al. 2010), a Markov Chain Monte Carlo (MCMC) chain length of 10000000 generations, a subsampling frequency of every 4000 generations, the first 100000 generations were discarded as burn-in, four heated chains and a chain temperature parameter of 0.2. Calculations were performed on the UBELIx (http://www.id.unibe.ch/hpc), the HPC cluster at the University of Bern.

The single gene alignments of COI, 16S, and ITS2 were imported into MEGA X 10.1.7 (Kumar et al. 2018) and the various sequences grouped into species. The average evolutionary divergence between sequence pairs within species (subsequently referred to as within-species divergence) was estimated where possible (only for species with more than one sequence present) using the Maximum Composite Likelihood model (Tamura et al. 2004) on standard settings. The Maximum Composite Likelihood model was also used to estimate the average evolutionary divergence between sequence pairs between species (subsequently referred to as between-species divergence). The focus of the analyses lay on the *Z.pretneri* group (as defined by Inäbnit et al. 2019; all markers) and the *Z.alpestre* group (only COI, with the Austrian specimens from Kruckenhauser et al. 2019) classified as separate species or included in *Z.amoenum*.

Additionally, an Automatic Barcode Gap Discovery (**ABGD**; Puillandre et al. 2011; https://bioinfo.mnhn.fr/abi/public/abgd/abgdweb.html) analysis was performed on the COI alignments of the *Z.pretneri* group and of the *Z.alpestre* group using the default settings (Pmin = 0.001, Pmax = 0.1, Steps = 10, X = 1.5, Nb bins = 20, distance = Jukes-Cantor).

A map (Fig. 1) was constructed using the Natural Earth dataset in QGIS 3.16.3. Most locality data was taken from Inäbnit et al. (2019), and the coordinates for the Austrian sites were taken from Kruckenhauser et al. (2019). Locality data of the specimens sequenced in this study were provided by the various collectors.

## Results

### Phylogenetic trees

**Figure 2.**
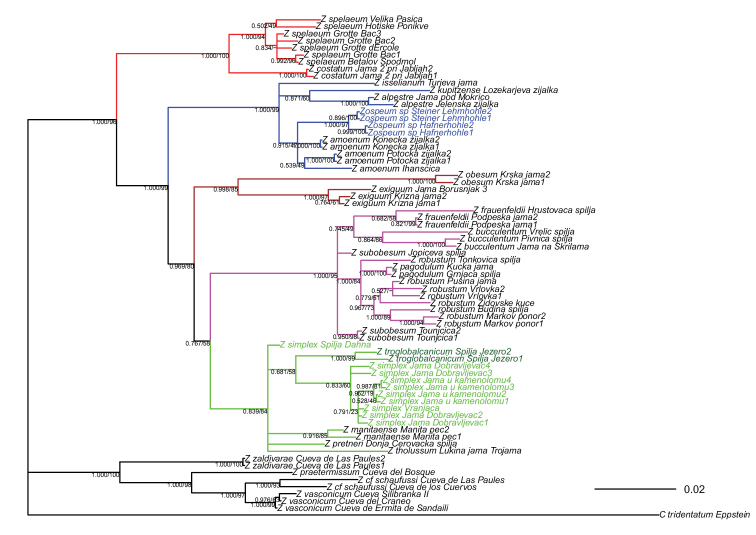
Bayesian tree of the genus *Zospeum*. Node support values of both the Bayesian Inference (front) and the Maximum Likelihood analysis (back) are given. Branches are coloured to denote the informal species groups within the eastern radiation of *Zospeum* following Inäbnit et al. (2019). Coloured sample names indicate specimens not included in the tree in Inäbnit et al. (2019): blue: Austrian specimens from Kruckenhauser et al. (2019); dark green: *Zospeumtroglobalcanicum*; light green: *Zospeumsimplex* sp. nov.

Both the ML and the BI trees (see Fig. 2 for the latter) are more or less identical. The specimens sequenced in this study clustered with *Z.pretneri*, *Z.tholussum*, and *Z.manitaense*. In both trees they form a badly supported monophyletic group that splits again into two groups in accordance with their geographical distribution (see Fig. 1) and could be separated at the species level: the two specimens from the region of Dubrovnik, Croatia (Špilja Jezero; referred to as Z.aff.troglobalcanicum), and the remaining specimens from Bosnia-Herzegovina (Jama u kamenolomu, Vranjača, Jama Dobravljevac; described as *Z.simplex* sp. nov. herein). The latter group is not supported in either tree but recovered in both. An additional specimen (NMBE 568054, Špilja Dahna), from which we were only able to amplify H3, didn’t cluster with any species within the *Z.pretneri* group. The two groups were also recovered, though here with high node support, in the additional ML tree (Suplementary tree 1) calculated for the *Z.pretneri* group. The Austrian specimens from Kruckenhauser et al. (2019) form a strongly supported monophyletic group within *Z.amoenum*.

### Divergences

For most markers, intraspecific divergences among the species in the *Z.pretneri* group are clearly smaller than the interspecific divergences (Table 3). This indicates that these species comprise separate lineages, especially the specimens classified as Z.aff.troglobalcanicum and those collected in Bosnia (henceforth referred to as *Z.simplex* sp. nov.), which were not included in previous genetic studies (see Inäbnit et al. 2019).

**Table 3.** The number of base substitutions per site from averaging over all sequence pairs within (within-species divergences) and between (between-species divergences) species within the *Z.pretneri* group. Results shown for each marker separately. Between-species distances are listed below the black, empty boxes, the Standard errors above.

COI										
Species	No. of sequences	Within-species divergences		Between-species divergences						
		Divergence	Standard Error	*Z.tholussum*	*Z.pretneri*		*Z.manitaense*	*Z.simplex* sp. nov.		Z.aff.troglobalcanicum
*Z.tholussum*	1	—	—		0.0126		0.0152	0.0148		0.0142
*Z.pretneri*	1	—	—	0.0602			0.0123	0.0148		0.0123
*Z.manitaense*	1	—	—	0.0849	0.0618			0.0161		0.0167
*Z.simplex* sp. nov.	9	0.0034	0.0018	0.0765	0.0779		0.0882			0.0133
Z.aff.troglobalcanicum	2	0.0288	0.0078	0.0777	0.0628		0.0974	0.0724		
**16S**										
Species	No. of sequences	Within-species divergences		Between-species divergences						
		Divergence	Standard Error	*Z.pretneri*		*Z.manitaense*			*Z.simplex* sp. nov.	
*Z.pretneri*	1	—	—			0.0079			0.0097	
*Z.manitaense*	2	0.0045	0.0031	0.0302					0.0078	
*Z.simplex* sp. nov.	9	0.005	0.0022	0.0389		0.0301				
**ITS2**										
Species	No. of sequences	Within-species divergences		Between-species divergences						
		Divergence	Standard Error	*Z.simplex* sp. nov.		*Z.manitaense*			Z.aff.troglobalcanicum	
*Z.simplex* sp. nov.	8	0.012	0.003			0.0055			0.0056	
*Z.manitaense*	1	—	—	0.0226					0.0074	
Z.aff.troglobalcanicum	2	0.0072	0.0035	0.0219		0.0278				

*Zospeumamoenum* shows a high intraspecific divergence when compared to other members of the *Z.alpestre group* (see Table 4), though other species (such as Z.aff.troglobalcanicum, see Table 3) show similarly high intraspecific divergence. When the Austrian populations from Kruckenhauser et al. (2019) are aligned within *Z.amoenum*, the interspecific divergence within the *Z.alpestre* group ranges between 0.0564–0.067. The between-group divergence amongst *Z.amoenum* sensu Inäbnit et al. (2019) and the specimens from Kruckenhauser et al. (2019) was smaller (0.0348±0.0071) than that amidst the other species within the *Z.alpestre* group, but still higher than the within-group divergence in both *Z.amoenum* and the Austrian specimens.

**Table 4.** The number of base substitutions per site from averaging over all sequence pairs within (within-species divergences) and between (between-species divergences) species within the *Z.alpestre* group for the marker COI. Shown are results, where the four Austrian specimens were considered a separate species and where the Austrian specimens were considered conspecific with *Z.amoenum*. Between-species distances are listed below the black, empty boxes, the Standard errors above.

Austrian populations treated as a separate species											
Species	No. of sequences	Within-species divergences		Between-species divergences							
		Divergence	Standard Error	*Z.amoenum*	Austrian pops.		*Z.alpestre*		*Z.isselianum*		*Z.kupitzense*
*Z.amoenum*	5	0.0203	0.0048		0.0071		0.0105		0.0104		0.0118
Austrian pops.	4	0.0062	0.0026	0.0348			0.0117		0.0107		0.0126
*Z.alpestre*	2	0.0098	0.0039	0.0564	0.0629				0.0133		0.013
*Z.isselianum*	1	—	—	0.0554	0.0524		0.0693				0.0131
*Z.kupitzense*	1	—	—	0.067	0.0704		0.075		0.0718		
**Austrian populations included in Z.amoenum**											
Species	No. of sequences	Within-species divergences		Between-species divergences							
		Divergence	Standard Error	*Z.amoenum*		*Z.alpestre*		*Z.isselianum*		*Z.kupitzense*	
*Z.amoenum*	9	0.02599	0.0055			0.0109		0.0099		0.0112	
*Z.alpestre*	2	0.0098	0.004	0.0593				0.013		0.0129	
*Z.isselianum*	1	—	—	0.0541		0.0693				0.0127	
*Z.kupitzense*	1	—	—	0.0685		0.075		0.0718			

### Automatic Barcode Gap Discovery (ABGD)

The ABGD run on the *Z.pretneri*-group COI alignment yielded two different possible subdivision schemes: one where the alignment was subdivided into five groups (five groups scheme; prior maximal distance P = 7.74e^-03^; barcode gap distance: 0.043) and a second where the alignment was subdivided into seven groups (seven groups scheme; prior maximal distance P = 4.64e^-03^; barcode gap distance: 0.003). Both subdivision schemes considered the previously published sequences of *Z.pretneri*, *Z.tholussum*, and *Z.manitaense* as separate groups. The five-group scheme separated the individuals sequenced in this study into a Croatian group (Špilja Jezero) and a Bosnian group (Jama Dobravljevac, Jama u kamenolomu, Vranjača), while the seven-group scheme separated those individuals into two Croatian groups (one for each of the two specimens from Špilja Jezero) and two Bosnian groups (1: specimens from Jama u kamenolomu; 2: specimens from Jama Dobravljevac and Vranjača).

The ABGD run on the *Z.alpestre*-group COI alignment yielded one subdivision scheme with seven groups (prior maximal distance P = 4.64e^-03^; barcode gap distance: 0.016): *Z.isselianum*, *Z.alpestre*, *Z.kupitzense*, *Z.amoenum* from Ihanščica, *Z.amoenum* from Konečka zijalka, *Z.amoenum* from Potočka zijalka and *Zospeumsp.* from Austria.

### Taxonomic implications

#### Zospeum simplex

Taxon classificationAnimaliaEllobiidaEllobiidae

Inäbnit, Jochum & Neubert, sp. nov.

04D6F12E-943D-5710-A586-2DFCA1508456

http://zoobank.org/0B924616-1AC1-49B8-BE5F-531286EACE63

Figures 1 , 3

##### Type specimens.

***Holotype*:**NMBE 568060, Jama Dobravljevac, 25.08.2019, leg. R. Slapnik & J. Valentinčič; ***Paratypes***: NMBE 568061–568063; SMF 349425, 4 shells; RSC 3760, 6 shells; Jama Dobravljevac, 25.08.2019, leg. R. Slapnik & J. Valentinčič.

##### Specimens examined.

NMBE 568054, Špilja Dahna, 03.09.2009, leg. A. Schoenhoffer; NMBE 568055–568058, Jama u kamenolomu, 24.08.2019, leg. R. Slapnik & J. Valentinčič; NMBE 568059, Vranjača, 24.08.2019, leg. R. Slapnik & J. Valentinčič.

##### Diagnosis.

Shell usually ca. 1.3 mm in height, transparent, conical, peristome thickened, roundish, with a differentiated parietal shield, lamellae not present.

Measurements (n = 9): Shell height: 1.26–1.42 mm (mean: 1.378 ± 0.047 mm); shell width: 0.93–1.04 mm (mean: 0.976 ± 0.035 mm); aperture height: 0.54–0.67 mm (mean: 0.6 ± 0.037); aperture width: 0.54–0.65 mm (mean: 0.601 ± 0.033 mm); number of whorls: 5–5.5.

##### Description.

Shell conical, translucent when fresh; suture deep; aperture somewhat roundish to reniform; parietal shield clearly differentiated from the rest of the lip, straight and thin; no lamellae present.

Differing from *Z.pretneri* and *Z.tholussum* by its broader shell and the differentiated parietal shield; differs from *Z.manitaense* by the absence of a visible parietalis in the aperture; barely differs from Z.aff.troglobalcanicum morphologically, on average with reduced shell broadness and a slightly deeper suture (see Remarks).

##### Distribution.

Known from four caves (Jama Dobravljevac, Špilja Dahna, Jama u kamenolomu, Vranjača) in the municipality of Tomislavgrad in Bosnia-Herzegovina.

##### Etymology.

Named *simplex* (= simple, unsophisticated) due to the lack of any form of shell sculpture or lamellae.

##### Remarks.

**Figure 3.**
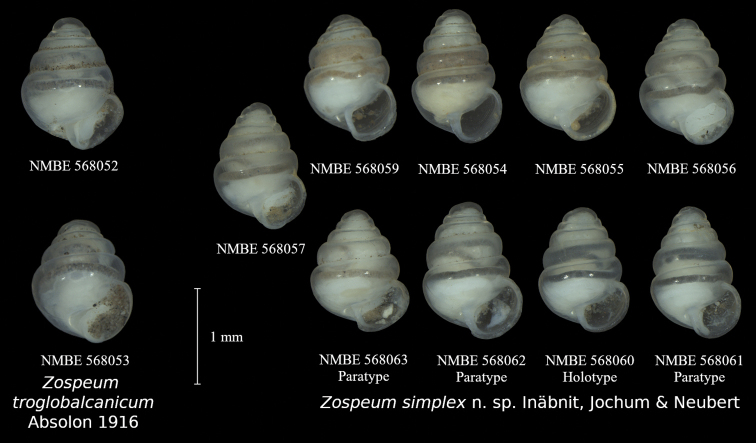
Specimens sequenced in this study. *Zospeumtroglobalcanicum*: NMBE 568052 & 568053 (both from Špilja Jezero); *Zospeumsimplex* sp. nov.: NMBE 568054 (Špilja Dahna), NMBE 568055–568057 (Jama u kamenolomu), NMBE 568059 (Vranjača), NMBE 568060 (Holotype, Jama Dobravljevac), NMBE 568061–568063 (Paratypes, Jama Dobravljevac)

Difficult to separate from *Z.troglobalcanicum* without genetic data (which is not uncommon in *Zospeum*; see Inäbnit et al. 2019). Both species have a nondescript shell without prominent shell sculpture or lamellae within the aperture. Absolon’s (1916) description of *Z.troglobalcanicum* consisted out of a photograph depicting multiple specimens haphazardly clustered together in various positions and a legend that established the name and type locality. The lack of a written characterisation of the species in the original description and the fact that the specimens in the photograph weren’t depicted in any standardised position makes a characterisation of the species fairly challenging (putative syntype specimen, collected by K. Absolon from the type locality, was only located very recently by AJ in Vienna (NHMW Mol.Coll.Edlauer 32.749) and couldn’t be studied yet). From the photograph in Absolon (1916), the species can be characterised as similar to *Z.manitaense* in shell shape, without any visible lamella in the aperture and with a comparatively large parietal shield. The larger parietal shield might serve as a distinguishing character between *Z.simplex* and *Z.troglobalcanicum*, though the illustration of a topotypic specimen in Bole (1974; fig. 3h) might indicate that this character is variable within the population. The two specimens we preliminarily assigned to *Z.troglobalcanicum* (Fig. 3, NMBE 568052; Inäbnit et al. 2019: fig. 7u) only have a small parietal shield. As of now, the shell height:shell width ratio seems to be the most effective way of separating the two specimens from *Z.simplex* (*Z.simplex*: generally higher than 1.3 (one exception); Z.aff.troglobalcanicum: below 1.3), but that might just be due to the low sample sizes. Investigation of the inner aspects of the shells will be presented in a later work.

## Discussion

The phylogenetic tree reconstructions (Fig. 2) agree mostly with those figured in Inäbnit et al. (2019). The main difference is that the node support values within the *Z.pretneri* group and in that of *Z.amoenum* are now fairly low and the topology is different. This can be explained by the high number of new specimens that sometimes are only represented by one marker (especially in *Z.amoenum*). It should also be noted that our current trees resolve *Z.robustum*, for which we didn’t have any new specimens, with a significant node support as a monophyletic group (node support was not significant in Inäbnit et al. 2019, but the classification as an independent species could be justified via species delimitation methods). Since its position was not resolved with significant node support in either tree, the specimen from Tonkovića špilja is not included in *Z.robustum* in this tree, as was the case in Inäbnit et al. (2019). Due to lack of additional material, the classification within *Z.robustum* remains unchanged in this work.

The 12 *Zospeum* individuals from Bosnia-Herzegovina and Croatia, are the first to be molecularly assessed from the greatly understudied, southern extension of *Zospeum*’s distribution. Within the phylogenetic trees (Fig. 2, Suppl. material 1), these specimens form a monophyletic group with a deep split between the two specimens from Croatia and nine of the ten specimens from Bosnia-Herzegovina (the remaining specimen from Špilja Dahna is only represented by a sequence of the conservative histone H3 gene, which doesn’t usually resolve to species level).While recovered in all phylogenetic trees calculated for this work, this arrangement only has high node support values in the Suppl. material 1, which was calculated without the conservative H3 and 28S nuclear markers. This result might indicate that conservative markers may have a destabilising effect on species level phylogeny within this group. Both ABGD schemes support the separation of the Croatian and Bosnia-Herzegovina individuals from each other at species level, though the seven-group scheme further subdivided the specimens from both geographical regions. We prefer to use the five-group scheme for the following reasons here: a) The barcode gap of the seven-group scheme is much lower (0.003) than the barcode gap (0.032) that was detected in the Carychiidae alignment in Weigand et al. (2011), while the barcode gap in the five-group scheme was slightly higher (0.043) than in Weigand et al. (2011); b) both individuals from Croatia (considered separate groups in the seven-group scheme) derive from the same cave and are unambiguously recovered as monophyletic and closely related in all trees, making their status as separate taxa unlikely. The divergence analysis further corroborates the results of the ABGD five-group scheme whereby the between-group divergence between the Croatian and the Bosnian groups (see Table 3) was within the general range of interspecific divergence within the *Z.pretneri* group. We thus, propose separating the individuals sequenced in this study into two species:

- A species encompassing all ten specimens from Bosnia-Herzegovina. This species is described as Z. simplex sp. nov. above. Since we do not have enough molecular and morphological data for the individual from Špilja Dahna, we cannot confidently place it within Z. simplex right now. However, due to its close geo graphical proximity (less than 1 km) to one of the caves with genetically identified specimens (Jama u kamenolomu), we expect it could well be assignable to Z. simplex as no external morphological inconsistencies separate it from other Z. simplex specimens in our study.
- A species comprising two specimens from Špilja Jezero in the region of Dubrovnik. This locality is fairly close (around 22 km) to the type locality (Benetina pećina) of Z. troglobalcanicum Absolon, 1916. The sequenced specimens do not show any major external morphological differences from the specimen identified as Z. troglobalcanicum (as figured in Bole 1974: fig. 3h) and from those imaged in Inäbnit et al. 2019: fig. 7u), though the adult specimen clearly has a smaller parietal shield than the specimens figured in Absolon (1916). We propose tentatively classifying those specimens within Z. troglobalcanicum until genetic material from the type locality can clarify its status and the morphological investigation of the singular syntype (NHMW Mol.Coll.Edlauer 32.749) of this species can be taxonomically and nomenclaturally clarified in a separate work.

Even if it is not as large as the between-group divergence of other species pairs within the *Z.alpestre* group, our divergence analysis revealed that the between-group divergence between *Z.amoenum* and the two Austrian populations is greater than the within-group divergence of either lineage. Our analysis also found that the within-group divergence in *Z.amoenum* is only slightly increased if the Austrian populations are included within this species. These results agree with the tree reconstruction published in Kruckenhauser et al. (2019), which resolved the Austrian population as the sister group of *Z.amoenum*. Our trees, as mentioned above, lack the resolution to separate the Austrian populations from *Z.amoenum* and can thus, not confirm this conclusion. The ABGD scheme for the *Z.alpestre* group recovers the Austrian population as a separate group from *Z.amoenum* and splits the latter species into three groups. The barcode gap in this scheme is, however, much lower (0.016) than the one proposed for Carychiidae in Weigand et al. (2011), which was used for species classification within the *Z.alpestre* group before (e.g., in Weigand et al. 2013). We are thus, reluctant to draw conclusions regarding *Z.amoenum* and the Austrian specimens from the ABGD scheme. It may indicate some large intraspecific genetic variability within *Z.amoenum* (with the possibility of the presence of several species) that might coincide with the large morphological variation found in this species (Inäbnit et al. 2019), which would need to be addressed in a separate study with better sampling.

*Zospeumamoenum* described in Inäbnit et al. (2019) bears either a small parietalis that does not expand within the shell or it is lacking completely. Kruckenhauser et al. (2019) did not figure a specimen in which the configuration of the parietalis within the last whorl could be seen, but Gittenberger (1982) figured one specimen from the Hafnerhöhle (one of the two caves sampled by Kruckenhauser et al. 2019), where the parietalis was exposed. The parietalis of this specimen is slightly broadened three quarters of a whorl into the shell and seems to decrease expansion again further into the shell. Though the syntype of *Z.amoenum* (see Inäbnit et al. 2019: fig. 6L) shows a similar configuration of the parietalis, it is not congruent with the description of this structure in *Z.amoenum* assessed in Inäbnit et al. (2019).

Our study suggests that a final species assignment for the two Austrian populations is not possible until further supporting information becomes available. Until then, we classify these two Austrian populations as *Z.amoenum*, avoiding the now outdated classification of these populations with *Z.isselianum* (as was done in Kruckenhauser et al. 2019).

## Supplementary Material

XML Treatment for Zospeum simplex

## Supplementary materials

Supplementary material 1 Figure S1 Data type phylogenetic tree https://binary.pensoft.net/file/612374This dataset is made available under the Open Database License (http://opendatacommons.org/licenses/odbl/1.0/). The Open Database License (ODbL) is a license agreement intended to allow users to freely share, modify, and use this Dataset while maintaining this same freedom for others, provided that the original source and author(s) are credited.Thomas Inäbnit, Adrienne Jochum, Raijko Slapnik, Eike Neubert
