# Supplementary figures and images for: New genetic data reveals a new species of Zospeum in Bosnia (Gastropoda, Ellobioidea, Carychiinae)
Source: Zookeys. 2021 Nov 18;1071:175–93. doi: 10.3897/zookeys.1071.66417 (PMC8616889; doi:10.3897/zookeys.1071.66417)

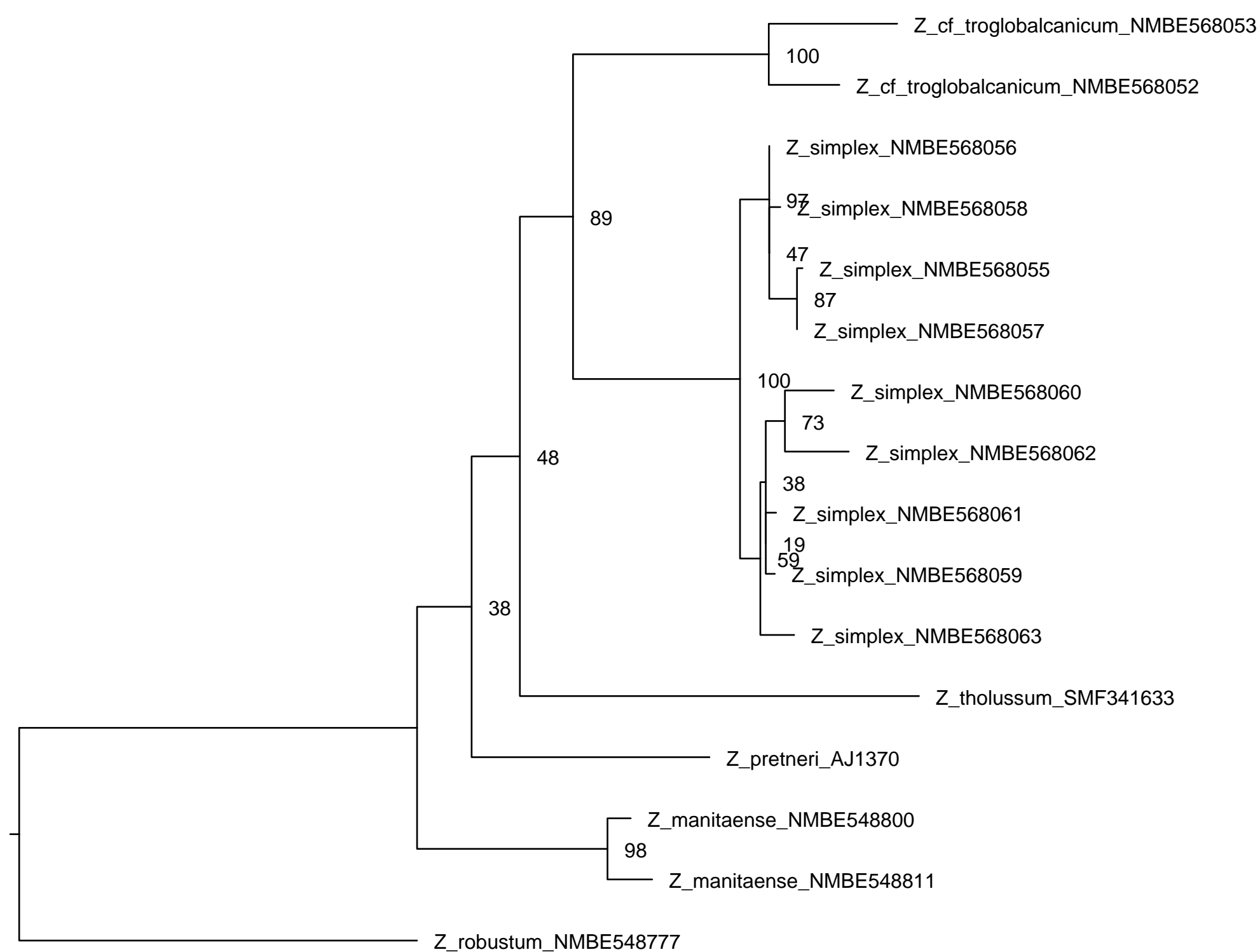

0.02

Supplement: Supplementary material 1 — Figure S1 [file zookeys-1071-175-s001.pdf]
